# Early hyperaesthesia and late hypoaesthesia as manifestations of sensory neuropathy in dentistry – two case reports

**DOI:** 10.3389/fendo.2026.1741118

**Published:** 2026-01-28

**Authors:** Laura Lipták, Klaudia Lipták, Péter Kempler, Péter Hermann, Noémi Katinka Rózsa, Gergely Balaton, Dániel Végh, Adrienn Menyhárt, Anna Körei, Dóra Marietta Balogh, Dorottya Bányai

**Affiliations:** 1 Department of Paediatric Dentistry and Orthodontics, Semmelweis University, Budapest, Hungary; 2 Department of Prosthodontics, Semmelweis University, Budapest, Hungary; 3 Department of Internal Medicine and Oncology, Semmelweis University, Faculty of Medicine, Budapest, Hungary

**Keywords:** case report, diabetes mellitus, oral complications, paediatric dentistry, sensory neuropathy

## Abstract

Diabetic neuropathy may present with a wide spectrum of sensory manifestations in the oral cavity, ranging from increased sensitivity to pain to impaired nociception. Early detection is crucial, since the changes have significant effects on dental treatment, patient safety and quality of life. We describe two contrasting pictures of patients with diabetes mellitus with signs of oral sensory neuropathy. The first case was a 14-year-old female with poorly controlled type 1 diabetes mellitus (T1DM) presenting with chronic carious lesions, gingivitis, and angular cheilitis. She also had amplified pain reactions to dental procedures despite adequate local anaesthesia and quantitative sensory testing revealed hyperaesthesia related to early diabetic neuropathy. The second patient was a 55-year-old male with a long-term history of type 2 diabetes mellitus (T2DM) with chronic periodontitis and reduced oral sensitivity. He complained of hypoesthesia and reduced pain perception during dental procedures with evidence of established neuropathic involvement. These cases illustrate the broad spectrum of clinical manifestations of diabetic sensory neuropathy in stomatology from early hyperaesthesia in a child with T1DM to late hypoesthesia in an adult with T2DM and illustrate the importance of metabolic control, interdisciplinary collaboration and individualised diagnostic and therapeutic strategies in dentistry.

## 1 Introduction

Type 1 diabetes mellitus (T1DM) represents one of the most frequent chronic conditions in childhood and adolescence, with incidence rates steadily rising across the globe. According to the International Diabetes Federation’s Diabetes Atlas, an estimated 1.0 million children aged 0–14 years were living with type 1 diabetes globally in 2025 (1). In Hungary, the prevalence is estimated to be approximately 1 in 600 children (2). While T1DM dominates in the paediatric population, type 2 diabetes mellitus (T2DM) accounts for the majority of cases in adults and represents a major global health burden, affecting more than 90% of people with diabetes worldwide (3). Beyond the well-established systemic complications, both T1DM and T2DM carry significant oral health implications, which can markedly affect patients’ quality of life and long-term prognosis (4–6).

The spectrum of oral manifestations is broad: in children with T1DM, accelerated tooth eruption (7, 8), higher caries risk, gingival inflammation, periodontal disease, oral mucosal alterations such as ulcerations and angular cheilitis (9, 10) and reduced salivary secretion (11) have all been documented. In adults with T2DM, periodontal disease is particularly prevalent and severe, with increased susceptibility to infections, impaired wound healing, and higher tooth loss rates (5, 6, 9). These findings underscore the bidirectional relationship between hyperglycaemia and periodontal pathology, which is now recognized as a major link between oral and systemic health (12). Despite this evidence, oral complications are frequently under-recognized compared with other diabetic sequelae such as ophthalmic, renal, and cardiovascular involvement (7, 12). This underestimation has been highlighted particularly in T2DM populations, where dental manifestations can remain untreated for years and contribute to systemic inflammatory burden (7).

Neuropathy is among the most serious microvascular complications of diabetes and, while most frequently studied in adults with T2DM, it may emerge earlier than previously assumed, even in adolescents with poorly controlled T1DM (13–16). In the orofacial region, neuropathic alterations may manifest as paradoxical pain amplification (hyperaesthesia, hyperalgesia, allodynia) (4, 17–19) or as decreased nociception (hypoesthesia, numbness). Such abnormalities can profoundly influence dental treatment, particularly in relation to anaesthetic effectiveness, pain perception, and patient cooperation (20, 21).

Severe oral complications such as carious lesions, gingivitis, and angular cheilitis in children with T1DM may in some cases be accompanied by hyperaesthesia verified by quantitative sensory testing (13). By contrast, late-stage presentations with diminished or absent oral pain sensation are rarely documented in the literature, particularly in adults with long-standing T2DM (22).

The novelty of the present report lies in presenting two contrasting manifestations of sensory neuropathy in diabetic patients: a 14-year-old girl with T1DM who exhibited exaggerated pain responses and hyperaesthesia, and a 55-year-old man with T2DM who demonstrated diminished sensitivity and hypoesthesia. By juxtaposing both ends of the neuropathic spectrum within the stomatological context, this study emphasizes the dynamic nature of sensory dysfunction in diabetes and its practical implications for dental care. These cases highlight the importance of early recognition, interdisciplinary collaboration between dentistry, neurology, and diabetology, and the need for individualized approaches to pain control in patients with diabetes.

## 2 Case 1: paediatric presentation

### 2.1 Case 1: description

A 14-year-old girl with a 10-year history of type 1 diabetes mellitus (T1DM) was admitted to the paediatric dental clinic with complaints of pain/and trismus in the region of the mandibular right second molar (tooth 47). She had been diagnosed with T1DM at the age of 4.5 years and she was receiving intensive insulin therapy in a basal–bolus regimen (Actrapid 7 U in the morning, 7 U at lunch, 8 U at dinner, and Levemir 18 U at bedtime, without insulin pump or sensor, administered via pen). Despite regular diabetological follow-up, her metabolic control remained poor, with a glycated haemoglobin (HbA1c) value of 11.1% at presentation. No systemic microvascular or macrovascular complications had yet been assessed.

**Figure 1:**
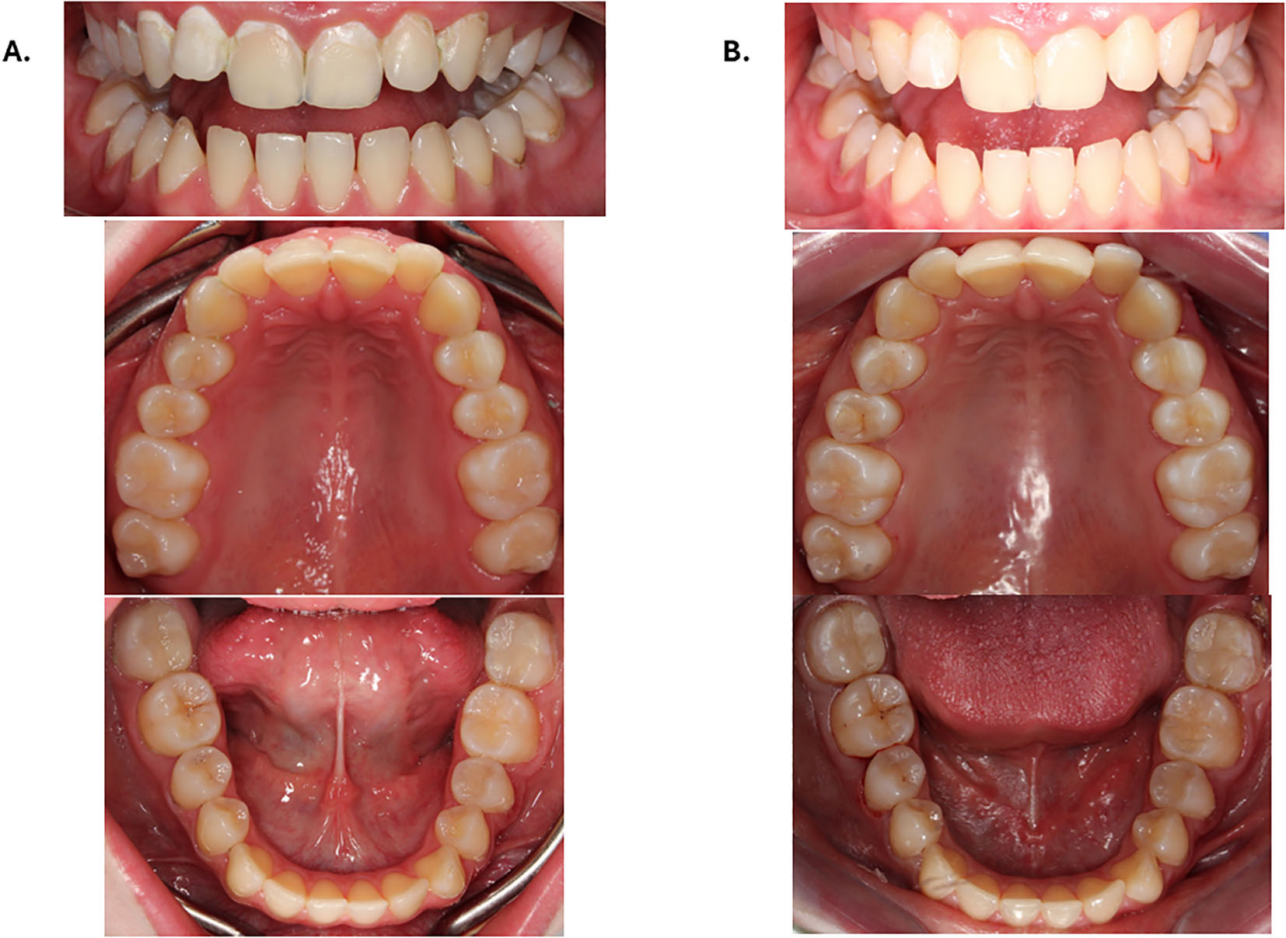
**(A)** Initial intraoral condition; **(B)** Intraoral photographs after dental rehabilitation.

Clinical examination revealed pericoronitis at tooth 47, generalized carious lesions, gingivitis, angular cheilitis, and poor oral hygiene with heavy plaque accumulation. According to the mother’s report, the patient often delayed dental visits due to anxiety and she complained of unusually severe pain during dental procedures even under local anaesthesia. Baseline intraoral photographs illustrated the overall oral condition, including generalized carious lesions and gingivitis, while pericoronitis at tooth 47 was identified based on clinical examination (**Figure 1A**).

### 2.2 Case 1: diagnostic assessment

The diagnostic evaluation included general physical examination, laboratory testing, and detailed dental and periodontal assessment, confirming poor metabolic control (HbA1c 11.1%) and extensive oral pathology. The patient reported disproportionately severe pain during dental procedures despite adequate local anaesthesia, which was inconsistent with the clinical extent of the dental findings and raised suspicion of altered sensory processing. Quantitative sensory testing (QST) of peripheral nerves demonstrated lowered sensory thresholds, indicating generalized sensory hyperexcitability. Intraoral hyperaesthesia was clinically inferred based on patient-reported symptoms and exaggerated pain responses during dental procedures, supported indirectly by the peripheral QST findings. The main diagnostic difficulty was to distinguish neuropathic pain from other possible causes, such as behavioural responses or anxiety-related hypersensitivity. Based on clinical and neurological findings, sensory hyperaesthesia as an early stage of diabetic sensory neuropathy was diagnosed.

### 2.3 Case 1: therapeutic intervention

Dental management focused on eliminating active caries, controlling gingival inflammation, and treating the acute pericoronitis at tooth 47. The inflamed operculum was managed, resulting in symptomatic relief. Multiple composite restorations were placed, and professional plaque removal was performed. Preventive strategies included oral hygiene instruction, topical fluoride application, and dietary counselling. During restorative procedures, the patient frequently required repeated administrations of local anaesthetic (articaine 4% with epinephrine 1:100,000 (Sanofi-Aventis Deutschland GmbH, Frankfurt am Main, Germany)); despite administration of three standard 1.8 ml cartridges (total dose approximately 216 mg), adequate analgesia was not achieved. Adjunctive behavioural management techniques were also applied to reduce dental anxiety and facilitate treatment. No sedation or systemic pharmacological therapy specific to neuropathy was administered.

### 2.4 Case 1: follow-up and outcomes

During the 12-month follow-up period, the patient required several additional restorative treatments due to recurrent caries. Despite repeated oral hygiene instructions, plaque accumulation and gingival inflammation persisted, indicating poor compliance with preventive measures. Professional plaque control and topical fluoride applications were regularly performed and well tolerated, but their effectiveness was limited by the patient’s inconsistent adherence to daily oral hygiene practices.

Throughout restorative procedures, behavioural management and repeated administrations of local anaesthetic were frequently necessary, as the patient continued to report disproportionately severe pain. Neurological follow-up, including quantitative sensory testing (QST), confirmed the presence of hyperaesthesia but did not demonstrate progression of neuropathy during this period.

No systemic diabetic complications were detected during the follow-up.

Follow-up intraoral photographs documented the outcomes of the restorative and preventive interventions (**Figure 1B**).

### 2.5 Case 1: patient perspective

The patient and her mother described dental visits as stressful due to the exaggerated pain sensation. Despite repeated or higher doses of anaesthetic, procedures were still perceived as unusually painful. During the procedure, a total of three cartridges of local anaesthetic were administered due to insufficient analgesic effect. They appreciated the supportive approach of the dental team, which facilitated treatment completion. The mother emphasized the importance of preventive strategies and regular follow-up, while acknowledging the everyday challenges of maintaining strict metabolic control.

### 2.6 Case 1: neuropathy assessment

Sensory polyneuropathy was assessed using the Neurometer device (Neurotron Inc., Baltimore, MD, USA), which is a quantitative sensory testing (QST) method that determines current perception thresholds (CPT) at three different frequencies (2000, 250, and 5 Hz). These frequencies selectively assess the function of large myelinated (Aβ), small myelinated (Aδ), and small unmyelinated (C) fibres, respectively. The technique allows for an objective evaluation of peripheral sensory nerve function and early detection of neuropathic changes. Measurements were performed on all four extremities. In Case 1, current perception threshold values of the right median nerve remained within the reference ranges across all tested frequencies. In contrast, CPT values obtained from the lower limb were below the lower limits of the reference ranges, indicating hyperaesthesia and suggesting early sensory involvement characteristic of diabetic neuropathy. This pattern is characteristic of early-stage diabetic neuropathy and corresponds with the exaggerated pain perception observed during dental procedures (**Table 1**). Cardiovascular autonomic function was evaluated using standard reflex tests. The deep-breathing test yielded borderline results, and orthostatic testing revealed a 12 mmHg drop in systolic blood pressure, consistent with orthostatic hypotension. The overall clinical presentation suggests an altered pain perception mechanism. While early carious lesions were associated with diminished pain sensation, dental procedures elicited disproportionately severe pain. This paradox can explain why multiple administrations of local anaesthetic did not provide adequate analgesia. Neuropathy-induced nerve fibre damage and altered nociceptive pathway function may lower anaesthetic efficacy and contribute to the marked interindividual variability observed in pain responses during dental treatment.

**Table 1:** Current perception thresholds of patient 1 as measured with the neurometer device.

Current perception thresholds (CPTs) (mA)						
Nervus medianus			Frequency (Hz)	Nervus peroneus		
Left	Reference range (mA)	Right		Left	Reference range (mA)	Right
250	120-398	350	2000 Hz	240	179-523	170
50	22-189	110	250 Hz	80	44-208	40
40	16-101	80	5 Hz	60	18-170	12

## 3 Case 2: adult presentation

### 3.1 Case 2: description

A 55-year-old man with a documented history of type 2 diabetes mellitus (T2DM) for more than 6 years presented to the dental clinic with aesthetic concerns regarding his dentition. His glycaemic control had long been poor, with glycated haemoglobin (HbA1c) values persistently above recommended targets and irregular attendance at diabetology follow-ups. His medical history included cardiovascular complications; no additional diabetic microvascular complications were documented at presentation.

Clinical examination revealed severe generalized periodontitis characterised by gingival recession, attachment loss, and tooth mobility. Multiple carious lesions were also recorded, yet the patient reported little or no pain, which was incongruent with the extent of pathology. He described diminished oral sensitivity during dental interventions and tended to underestimate the severity of his oral condition, which contributed to delays in seeking treatment.

**Figure 2:**
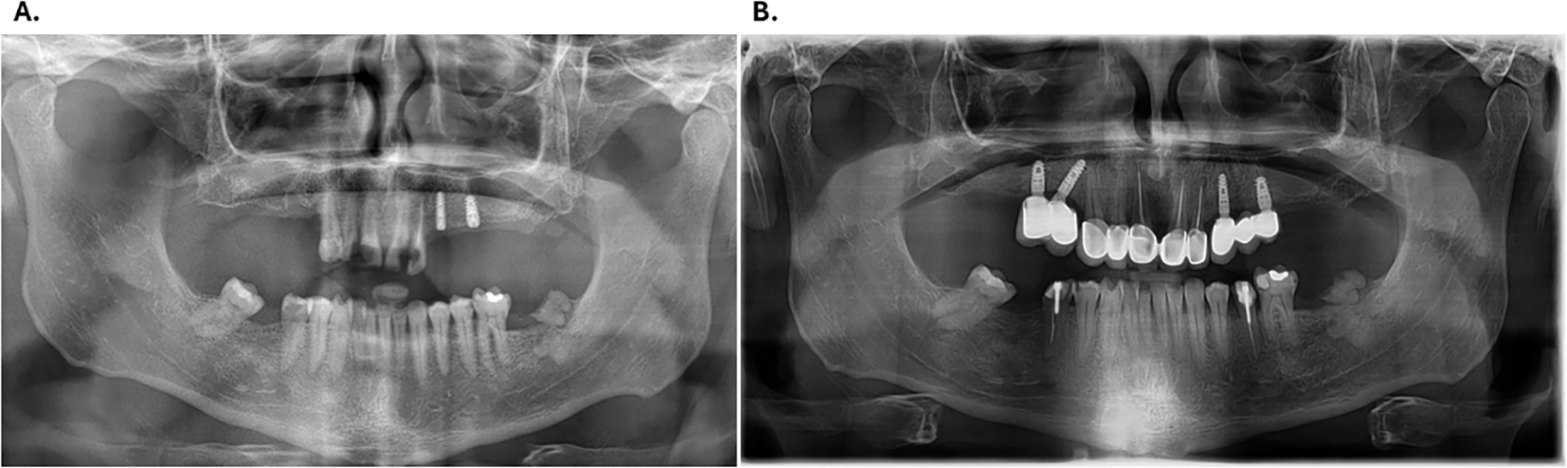
**(A)** Initial orthopantomogram; **(B)** Maxillary implants after the healing period.

A neurological consultation, including quantitative sensory testing (QST), confirmed hypoesthesia in oral and peripheral regions, consistent with advanced sensory neuropathy associated with long-standing diabetes. Baseline panoramic radiograph demonstrated the extent of carious and periodontal destruction (**Figure 2A**).

### 3.2 Case 2: diagnostic assessment

Routine laboratory testing confirmed poor metabolic control, with glycated haemoglobin (HbA1c) values persistently above recommended targets. Attendance at diabetology follow-ups was irregular, further contributing to the unstable glycaemic profile. Comprehensive dental and periodontal assessment revealed severe periodontitis with gingival recession, attachment loss, and tooth mobility, along with multiple untreated carious lesions. Despite the extent of pathology, the patient reported little or no pain, reflecting diminished oral sensitivity. Neurological evaluation, including quantitative sensory testing (QST), demonstrated hypoesthesia in both oral and peripheral regions. The main diagnostic difficulty was the absence of subjective complaints despite severe oral disease, which delayed recognition of both dental and neurological pathology. Based on the combined findings, a diagnosis of advanced diabetic sensory neuropathy was established.

### 3.3 Case 2: therapeutic intervention

Management primarily targeted the treatment of chronic periodontitis. Initial therapy included scaling and root planing combined with detailed oral hygiene instructions. Multiple composite restorations were placed to manage carious lesions, and endodontic treatment was performed on selected teeth due to advanced decay.

Local anaesthesia (articaine 4% with epinephrine 1:100,000 (Sanofi-Aventis Deutschland GmbH, Frankfurt am Main, Germany)) was administered for restorative and periodontal procedures. The patient reported little or no discomfort during treatment, which was consistent with his hypoesthesia. Because the absence of symptoms reduced his motivation for treatment, repeated education and encouragement were required to ensure adherence. Preventive measures included professional plaque control, topical fluoride applications, and chlorhexidine mouth rinses (0.12%; Haleon, Brentford, United Kingdom) administered in two-week courses. Close coordination with diabetology was also recommended to improve systemic control and reduce the risk of further progression. Following periodontal stabilization and restorative treatment, dental implants were placed in the maxilla. After the healing period, the final panoramic radiograph confirmed functional and aesthetic rehabilitation (**Figure 2B**).

### 3.4 Case 2: follow-up and outcomes

At the beginning of the follow-up period, patient motivation was weak, and compliance with both dental and diabetological recommendations remained poor. However, over time, his metabolic control gradually improved, which was accompanied by better oral hygiene practices. This positive change enabled further dental treatments, including the provision of fixed prosthetic restorations. All procedures were performed without pain due to hypoesthesia, and the absence of discomfort facilitated completion of complex interventions. Regular follow-up visits contributed to a gradual improvement in oral health and stabilization of periodontal status.

### 3.5 Case 2: patient perspective

The patient reported rarely experiencing discomfort during dental procedures and was surprised at the severity of his oral disease. He admitted that irregular diabetology attendance contributed to poor metabolic control. After counselling, he recognized the importance of more consistent medical and dental care. Although initially perceived as beneficial, the absence of pain ultimately delayed timely treatment.

### 3.6 Case 2: neuropathy assessment

Sensory neuropathy was evaluated using the Neurometer diagnostic device, which determined current perception thresholds on all four extremities. As shown in **Table 2**, CPT values of the left upper limb demonstrated sensory dysfunction, while the right upper limb exhibited elevated thresholds across all tested frequencies, indicating sensory fibre impairment. In the lower extremities, extremely severe abnormalities were detected: both sides showed maximal measurable values, reflecting profound dysfunction of the peroneal nerve fibres. Overall, the findings confirmed advanced diabetic sensory neuropathy, particularly affecting the lower limbs. Cardiovascular autonomic function was assessed by standard reflex tests, which demonstrated pathological alterations. The deep-breathing test yielded abnormal results, and a 24 mmHg drop in systolic blood pressure during orthostatic testing was consistent with orthostatic hypotension.

**Table 2:** Current perception thresholds of patient 2 as measured with the neurometer device.

Current perception thresholds (CPTs) (mA)						
Nervus medianus			Frequency (Hz)	Nervus peroneus		
Left	Reference range (mA)	Right		Left	Reference range (mA)	Right
650	120-398	600	2000 Hz	999	179-523	999
280	22-189	258	250 Hz	999	44-208	999
60	16-101	165	5 Hz	999	18-170	999

### 3.7 Summary of the two cases

Taken together, these cases illustrate two extremes of sensory neuropathy in diabetes: a 14-year-old girl with early hyperaesthesia and exaggerated pain responses, and a 55-year-old man with late hypoesthesia and markedly reduced pain sensation. In both patients, poor metabolic control was confirmed by elevated glycated haemoglobin (HbA1c) values, while clinical and neurological assessments verified abnormal sensory function. These findings provide the rationale for presenting the cases in detail and highlight the spectrum of neuropathic alterations relevant to dental management.

## 4 Timeline

The **Table 3** illustrates the progression of oral and neurological complications in the two cases. One patient exhibited early hyperaesthesia and recurrent dental problems, whereas the other showed advanced periodontal destruction, reduced oral sensitivity, and late-stage neuropathy.

**Table 3:** Comparison of oral and neurological alterations in two patients with diabetes.

Timepoint/age	Patient 1 (14-year-old female, T1DM) (23)	Patient 2 (55-year-old male, T2DM)
Age 4 – Patient 1 Age 49 – Patient 2	Diagnosis of type 1 diabetes mellitus. Poor glycaemic control from onset.	Diagnosis of type 2 diabetes mellitus in early adulthood.
Age 10–14 – Patient 1 Age 53 – Patient 2	Recurrent carious lesions, gingivitis, and angular cheilitis. Multiple restorations required.	Development of periodontal disease, progressive attachment loss. Suboptimal metabolic control.
Age 14 – Patient 1 Age 49 – Patient 2	Severe pain during dental care despite adequate local anaesthesia. Neurological exam confirmed hyperaesthesia.	Reduced oral sensitivity; pain-free despite active caries and severe periodontitis.
Age 14–present – Patient 1 Age 53 – Patient 2	Requires behavioural management and repeated local anaesthetic administration for dental treatment.	Neurological testing confirmed hypoesthesia; consistent with advanced diabetic neuropathy. Attendance at diabetology care is irregular.
Current status	Early sensory neuropathy with hyperaesthesia. Ongoing restorative and preventive care.	Late-stage neuropathy with hypoesthesia. Advanced periodontal treatment is required.

## 5 Discussion

Diabetic sensory neuropathy within the stomatognathic system can present at different stages of disease progression, with early hyperaesthesia in children and late hypoesthesia in adults. These alterations complicate both diagnosis and treatment, as they may reduce anaesthetic efficacy or mask advanced pathology. In paediatric patients, exaggerated pain responses and reduced efficacy of local anaesthesia may indicate early neuropathic involvement (17, 24–26). In contrast, in adults with long-standing diabetes, hypoesthesia may mask severe oral pathology, often delaying recognition until advanced stages (18).

In Case 2, before surgery, periodontal stabilization was achieved, and careful case selection was applied. Antibiotic prophylaxis was administered, and metabolic control was closely monitored in collaboration with the diabetology team. Nevertheless, implant therapy under such conditions remains controversial, as the literature reports increased risks of peri-implant complications and implant failure in poorly controlled diabetic patients. In Case 2, type 2 diabetes mellitus was diagnosed six years prior to presentation; however, the severity of sensory neuropathy and advanced oral complications suggests that the disease likely developed well before its clinical recognition. It is well established that type 2 diabetes often remains asymptomatic for prolonged periods, and microvascular complications typically arise only after sustained hyperglycaemia. This case, therefore, highlights the importance of individualized risk assessment and interdisciplinary decision-making.

This duality illustrates why neuropathic alterations represent a diagnostic challenge in dental practice. This case also underlines how oral hypoesthesia in adults with long-standing diabetes can mask destructive periodontal processes, leading to delayed presentation and treatment. Evidence supports that neuropathy is not confined to long disease duration in adults but can already be detected at a subclinical level in childhood and adolescence (15, 25, 26). On the other hand, sensory loss and hypoesthesia are well documented in adults with type 2 diabetes and frequently contribute to the delayed diagnosis of oral and periodontal disease (18, 22). Nevertheless, some studies, such as the pilot work by Körei et al. (27), found no clear evidence of neuropathy among patients at high risk for diabetes, highlighting the inconsistency of current data (27). The key clinical message from these cases is that unusual pain responses – whether increased or decreased – should raise suspicion of neuropathic involvement. Such findings justify interdisciplinary consultation with neurology and diabetology. Closer collaboration and individualized therapeutic strategies are essential to ensure safe and effective care of patients with diabetes mellitus in dental settings (14, 26). The systemic relevance of sensory neuropathy is further emphasized by its well-documented role in diabetic foot syndrome and other complications, underlining its importance beyond the oral cavity (28, 29).

## Data Availability

The original contributions presented in the study are included in the article/supplementary material. Further inquiries can be directed to the corresponding authors.
